# The Role of Screentime and Family Resiliency in Overweight/Obesity in Children and Children with Developmental Disabilities Before and During COVID-19

**DOI:** 10.3390/children13010066

**Published:** 2025-12-31

**Authors:** Purnima S. Mudnal, Emmeline Chuang, Olivia J. Lindly, Jack Needleman, David A. Ganz, Alice A. Kuo

**Affiliations:** 1 School of Public Health & Health Sciences, California State University, Dominguez Hills, Carson, CA 90747, USA; 2 Mack Center on Nonprofit and Public Sector Management in the Human Services, University of California, Berkeley, CA 94720, USA; emchuang@berkeley.edu; 3 Department of Health Sciences, Northern Arizona University, Flagstaff, AZ 86011, USA; olivia.lindly@nau.edu; 4 Department of Health Policy and Management, Fielding School of Public Health, University of California, Los Angeles, CA 90095, USA; needlema@ucla.edu (J.N.); akuo@mednet.ucla.edu (A.A.K.); 5 Department of Medicine, David Geffen School of Medicine, UCLA, Los Angeles, CA 90095, USA; 6 Center for the Study of Healthcare Innovation, Implementation and Policy, VA Greater Los Angeles Healthcare System, Los Angeles, CA 90073, USA

**Keywords:** pediatric obesity, overweight, child, family resilience, screen time, developmental disabilities, intellectual disability, COVID-19, children with special health care needs

## Abstract

**Highlights:**

**What are the main findings?**

- Prevalence of all children and children with developmental disabilities identified as overweight/obese reporting ≥4 h of screentime use increased from pre-COVID-19 to during COVID-19 years.
- Among all children, screentime was positively associated with overweight/obesity both before and during COVID-19, and associations with family resilience varied by resilience level and pandemic timepoint. For children with developmental disabilities, screentime associations and family resiliency were generally weaker across both pre- and during COVID-19.

**What are the implications of the main findings?**

- The rise in the proportion of children and children with developmental disabilities spending more than 4 h on screens during COVID-19 is an important factor to consider for childhood obesity interventions and policies.
- Family resiliency may play a protective role in preventing overweight/obesity and needs to be further investigated so that best practices in supporting children and families with early overweight/obesity screening can be paired with culturally sensitive programs.

**Abstract:**

Background/Objectives: This study examines factors associated with child overweight/obesity (OW/OB), pre-COVID-19 and during the COVID-19 pandemic, among all U.S. children aged 10–17 years, with or without developmental disabilities (DD) and, separately, among the subgroup of children diagnosed with a DD. Methods: Using data from the National Survey of Children’s Health (NSCH, 2018–2021), we applied descriptive statistics and multivariate logistic regression analyses to estimate the odds ratios of associations between family resilience, screen time, and childhood overweight/obesity. Family resilience measures families’ communication and problem-solving behaviors. Screentime is time spent on TV, computer, cellphone or electronic devices. Results: In descriptive analyses, during COVID-19, 35.8% of all children were identified as OW/OB compared to 32.8% pre-COVID-19—a weighted increase of 3.0%. Among children with developmental disabilities, OW/OB increased from 37.4% to 39.3%. Children reporting ≥4 h of screentime use increased from pre-COVID-19 to during COVID-19 in both groups (All Children: pre-COVID: 33.5%, during COVID: 41.6%; Developmental Disabilities: pre-COVID: 39.9%, during COVID: 49.4%). Among all children, there was a positive and strong association between screentime use and OW/OB at both pre- and during COVID-19 years. Children belonging to households with low family resiliency had 1.31 times the odds of being overweight/obese (95% CI, 1.06–1.63, *p* < 0.05) before the pandemic. However, these results were not significant after the pandemic. Conclusions: Prevalence of overweight/obesity in all children and children with DD during the COVID-19 pandemic continued to rise. Screentime was found to be a key determinant in increased weight status. Contrary to our hypothesis, family resilience failed to emerge as a significant protective factor for OW/OB; additional research is needed to explore the protective role of family resiliency on childhood obesity. Study findings may provide insights into developing best practices and tailored interventions with early OW/OB screening and programs tailored towards the youngest group of children aged 10–12 years or below.

## 1. Background

Over one third of U.S. children and adolescents are overweight or obese [1,2]. A child is generally considered overweight if their body mass index (BMI) falls within the 85th to 94th percentile range, while obesity is categorized as having a BMI in the 95th percentile or above [3]. Factors driving childhood overweight/obesity (OW/OB) may vary for children with special health care needs (CSHCN) and, specifically, for the subset of these children with developmental disabilities (DD).

A developmental disability is defined as a condition that impairs a child’s physical activity, learning, language, or behavior [4,5]. Developmental disabilities include conditions such as attention-deficit/hyperactivity disorder, autism spectrum disorder (ASD), blindness, cerebral palsy, moderate to profound hearing loss, learning disability, seizures, stuttering or stammering, and intellectual impairment [4,5]. More than one in six U.S. children (~17%) have one or more developmental disabilities and these individuals are two to three times more likely to experience obesity as compared to children without any special healthcare needs [6,7]. In general, CSHCN, and particularly those with developmental disabilities, are more prone to OW/OB due to several factors: prescription medication use for which weight gain is a side effect, genetic predisposition [8], physical and functional disabilities restricting physical activity, and more limited preventive weight screening [9,10].

Although the rate of childhood OW/OB approximately doubled during COVID-19 [11], few studies have examined trends in OW/OB specifically for children with developmental disabilities during the pandemic. This is a critical gap, as it is known that this subset of children with developmental disabilities faced reduced healthcare access and disruptions in therapy services during the pandemic [12] in addition to COVID-related school and work closures. These disruptions have led to a multitude of unfavorable conditions with respect to OW/OB, such as social isolation, parent- and child-related psychological stress, increased screentime, increased access to high-density foods/meals, and reduced or non-existent access to spaces that promote physical activity [13,14]. Given the pre-existing elevated risk of OW/OB among children with developmental disabilities, it is crucial to examine the impact of the pandemic on OW/OB in this subgroup of adolescents and to identify associated risk and protective factors.

These influential factors include myriad, multilevel determinants that can impact childhood OW/OB, including those at the individual, family, social and environmental levels [15]. For example, higher screentime is an established risk factor for OW/OB; children who spend one hour or more per day watching television or playing video games are more likely to be OW/OB [16,17]. Screentime is associated with lower physical activity and greater consumption of high-calorie foods in children [13,18,19,20]. On the other hand, family resiliency has been proposed as a potential protective factor in preventing OW/OB [21] and has been shown to positively affect children’s overall health, obesity and adverse childhood experiences [22,23]. Family resiliency in this work refers to the behaviors a family may employ to resolve household issues and may include approaches such as effective communication, drawing on family strengths, and maintaining hope [22]. Findings from recent studies have shown that family resiliency can play an important role in parental well-being and digital media use during the pandemic demonstrating that family resilience can affect family well-being as well as parental conflict over children’s digital media use [24,25,26]. Other factors, including age [22], limited access to green spaces [27], higher poverty levels [28], and a child’s race and ethnicity [28,29], have also been shown in prior work to be significantly associated with OW/OB.

In this work, we focus on screentime and family resilience as factors related to OW/OB for children and children with developmental disabilities and observe trends related to these factors during the pandemic. Specifically, we explore associations between these two factors and OW/OB status, pre-COVID and during the COVID-19 pandemic, among all U.S. children aged 10–17 years, and, separately, among the subgroup of children diagnosed with developmental disabilities. We hypothesized that both pre- and during the COVID-19 pandemic, there would be a positive association of screentime with OW/OB in all children and particularly those with developmental disabilities, after controlling for influential external factors. We additionally hypothesized that during both time periods, a negative association between family resilience and OW/OB would be observed for both groups of data.

## 2. Methods

This study is a secondary analysis of cross-sectional, publicly available National Survey of Children’s Health (NSCH) data from 2018 to 2019 (pre-COVID) and 2020–2021 (during COVID). The NSCH is a nationally representative annual survey of U.S. children ages less than 18 years, completed by parents and funded by the Health Resources and Services Administration. CSHCN and children under age five are oversampled (Child and Adolescent Health Measurement Initiative [30]. The population studied was children aged 10–17 years with and without developmental disabilities, with an additional subgroup analysis of those only with developmental disabilities. A child was considered to have a developmental disability if their caregiver answered “yes” to survey questions about whether the child had any neurodevelopmental conditions, including attention-deficit/hyperactivity disorder (ADHD), autism, learning disabilities, intellectual disability, conduct disorders, cerebral palsy, and impairments in vision and hearing [4,5].

Sample sizes and response rates from the 2018–2021 NSCH were as follows: 30,530 surveys completed with a weighted response rate of 43.1% in 2018; 29,433 surveys completed with a 42.4% response rate in 2019; 42,777 surveys completed with 42.4% response rate in 2020; and 50,892 surveys completed with a 40.3% response rate in 2021.

### 2.1. Measures

**Dependent Variable:** Childhood OW/OB was derived as a dichotomous variable according to body mass index (BMI) with values set = 0 for children that were normal weight (5th to <84th percentile) and set = 1 for those that were overweight (85th to 94th percentile) or obese (95th percentile or above). Underweight children (<5th percentile) were excluded from the analysis as we were interested in comparing normal-weight children with OW/OB children.

**Independent Variables**: Our main independent variables were screentime and family resiliency. Screentime was defined as time spent on TV, computer, cellphone, or other electronic devices from <1 h to 1.5 h per day, 2–3 h per day, and ≥4 h per day. Family resiliency was based on parental responses to four questions about whether the family demonstrates qualities of resiliency during difficult times. This variable measured how the family addressed problems by asking how likely they were to do at least one or more of the following behaviors: talk together, whether they work together to solve their problems, are they able to draw on family strengths, and staying hopeful in difficult times. Response options to each question were “none of the time”, “some of the time”, “most of the time”, or “all of the time.” Using responses to these questions, a family resilience composite variable comprising three levels was constructed. Levels included: low resiliency (an “all or most of the time” response to 0–1 items), medium resiliency (an “all or most of the time” response to 2–3 items), and high resiliency (an “all or most of the time” response to all 4 items).

**Covariates:** Covariates of interest included healthcare access, sociodemographic characteristics, environmental factors, and child health status. Healthcare access was assessed in terms of health insurance type and a composite variable measuring access to care that meets the medical home criteria as defined by the American Academy of Pediatrics [31]. The NSCH measures access to care meeting medical home criteria as having a usual source of care, having a personal physician or nurse, receiving family-centered care, receiving referrals for specialty care if needed, and receiving help coordinating health and health-related care if needed. Sociodemographic characteristics included household income, parental level of education, family structure, age, child race/ethnicity, and sex (assigned at birth). Child’s race/ethnicity was categorized as Non- Hispanic White, Non-Hispanic Black, Hispanic, Asian, American Indian/Alaskan Native, Native Hawaiian and Other Pacific Islander (AIAN, NHOPI). Environmental factors included whether a neighborhood had at least one, two, three, or four amenities and the food situation in each child’s household. Neighborhood amenities could include the following: having sidewalks or walking paths, parks/playgrounds, recreation or community centers (including boys’ and girls’ clubs), and/or having access to libraries or bookmobiles in the community. Household food situation was measured based on parental responses regarding their ability to afford sufficient food during the past 12 months. This variable included three levels reflecting the child’s household food situation: they always had nutritious food; always could afford to eat but not always nutritious; and sometimes or often could not afford enough to eat. Finally, health status was categorized based on parental reports as excellent/very good, good, or fair/poor.

### 2.2. Statistical Analysis

We computed weighted descriptive statistics to characterize the study sample of all children identified as OW/OB. Separate weighted descriptive analyses were also conducted for the OW/OB subgroup of children with only developmental disabilities.

We utilized weighted multivariate logistic regression models to estimate associations between our independent variables and being OW/OB, adjusting for previously defined covariates of interest. Models were run separately for children surveyed pre-COVID (2018–2019) and during the COVID-19 pandemic (2020–2021). These analyses were conducted for all children aged 10–17 years, as well as for the subgroup of children with developmental disabilities, resulting in a total of four models. Multivariate logistic regression results were visualized using forest plots, with separate plots created for all children and for the developmental disabilities subgroup.

Sensitivity and predictive margins analyses for the interaction between screentime and family resilience were subsequently conducted to assess whether overweight/obesity status varied across levels of these two factors. These findings were not statistically significant.

Statistical analyses were conducted using Stata statistical software, version 17 from Stata Corp. 2021, College Station, TX, USA and accounted for the complex survey design and imputation of income-related variables.

## 3. Results

During COVID, 35.8% of all children 10–17 years of age were identified as OW/OB (n = 35,368), compared to 32.8% pre-COVID—a weighted increase of 3.0% (Table 1). In both the pre- and during-COVID periods, the largest weighted percentage of all children with OW/OB was among those aged 10–12 years (Pre-COVID: 41.4%; During COVID: 42.5%). Among children with developmental disabilities, the weighted percentage with OW/OB increased from 37.4% to 39.3%, an increase of 1.9%.

Most children, regardless of whether diagnosed with a developmental disability, reported either 2–3 h or ≥4 h of daily screentime during both the pre-COVID and during COVID periods (Table 1). Furthermore, there was an increase in the percentage of children reporting ≥4 h of screentime use from pre-COVID to during COVID in both groups (All Children: pre-COVID: 33.5%, during COVID: 41.6%; Developmental Disabilities: pre-COVID: 39.9%, during COVID: 49.4%). High family resiliency was prevalent overall and increased among all children with OW/OB between pre-COVID (78.3%) and during COVID years (82.0%). Similar patterns were observed for the subgroup of children with developmental disabilities (pre-COVID: 70.4%; during COVID 74.9%).

**Figure 1:**
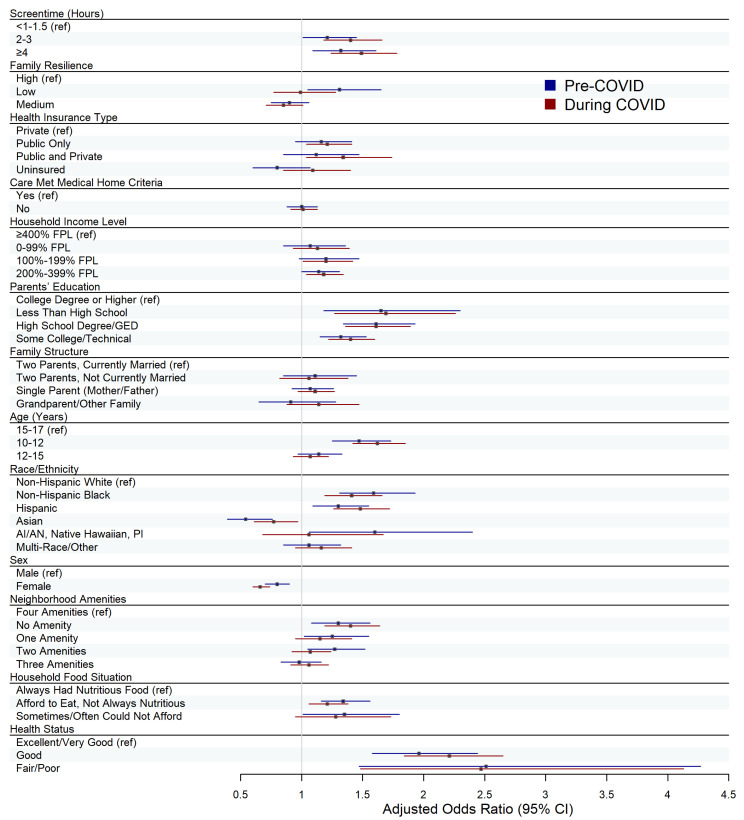
Forest Plot of Adjusted Weighted Logistic Regression Results for All Children. Weighted multivariate logistic regression models were fit to explore associations between screentime and OW/OB, and between family resilience and OW/OB, among all children (with or without developmental disabilities). Separate models were estimated for the pre-COVID period (Model 1) and the during-COVID period (Model 2), distinguished by color (blue = pre-COVID, red = during COVID). Boxes represent adjusted odds ratios, and horizontal lines represent 95% confidence intervals for each predictor, allowing comparison of associations across the two time periods. Reference groups are indicated with “(ref)” in parentheses.

Among all children, results of the weighted logistic regression models showed that at both pre- and during COVID, there was a positive association between screentime use and OW/OB (Figure 1; Table 2: Models 1 and 2). Relative to those with <1–1.5 h of screentime, children with ≥4 h of screentime had 1.32 (95% CI: 1.09, 1.61) times the odds of experiencing OW/OB pre-COVID, after adjusting for family resilience and covariates of interest. During COVID, this adjusted odds ratio (aOR) for all children with ≥4 h of screentime increased to 1.49 (95% CI: 1.24, 1.78). Both sets of findings were statistically significant (pre-COVID: *p* < 0.01; during COVID: *p* < 0.001). A similar pattern of increase in the adjusted odds of OW/OB from pre-COVID to during COVID was observed for children who had 2–3 h of screentime (pre-COVID: aOR = 1.21, 95% CI: 1.01, 1.45, *p* < 0.05; during COVID: aOR = 1.40, 95% CI: 1.18, 1.66, *p* < 0.001).

**Figure 2:**
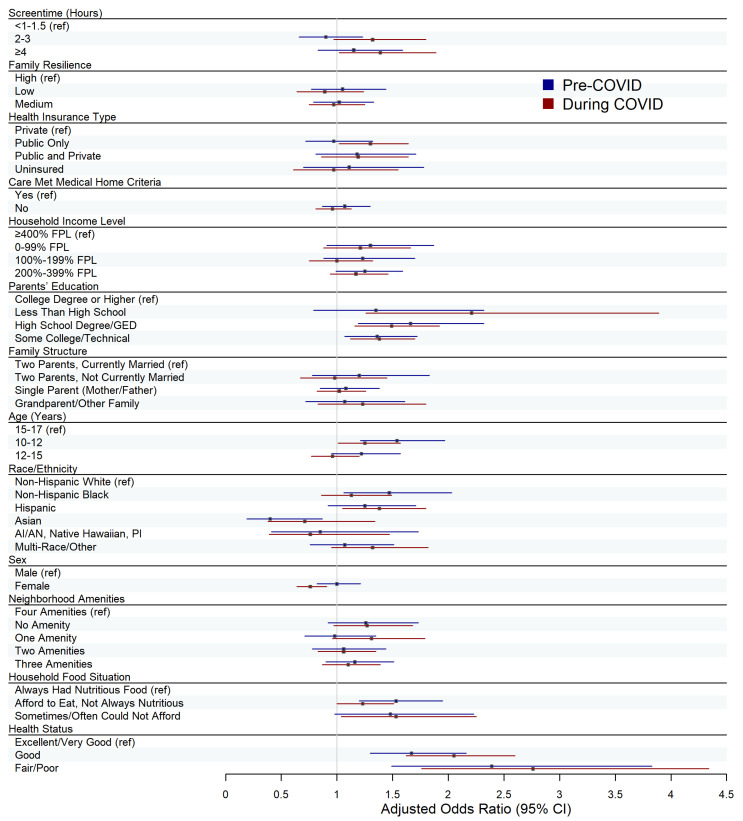
Forest Plot of Adjusted Weighted Logistic Regression Results for Children with Developmental Disabilities. Weighted multivariate logistic regression models were used to assess associations between screentime and OW/OB, and between family resilience and OW/OB, among the subgroup of children with developmental disabilities. Separate models were fit for the pre-COVID period (Model 3) and the during-COVID period (Model 4), and are distinguished by color (blue = pre-COVID, red = during COVID). Boxes represent adjusted odds ratios, and horizontal lines represent 95% confidence intervals for each predictor. Variable reference groups are indicated with a “(ref)” label.

Findings from the subgroup analysis of children with developmental disabilities demonstrated a generally weaker pattern of associations, with fewer statistically significant results compared to the sample of all children (Figure 2; Table 2: Models 3 and 4). Pre-COVID, the odds of being identified as OW/OB in children with ≥4 h of screentime were 1.15 (95% CI: 0.83, 1.59) times the odds of those with <1–1.5 h, after adjusting for family resilience and covariates of interest. These adjusted odds increased to 1.39 (95% CI: 1.02, 1.89) during the pandemic. The pre-COVID associations were not statistically significant (*p* ≥ 0.05), whereas those observed during COVID were (*p* < 0.05). After adjusting for family resilience and relevant covariates of interest, we also observed that pre-COVID, children with developmental disabilities with 2–3 h of screentime had lower odds of OW/OB compared to those with <1–1.5 h (aOR = 0.90, 95% CI: 0.66, 1.23), although this association did not reach statistical significance (*p* ≥ 0.05). During COVID, the adjusted odds shifted in a positive direction, indicating higher odds of OW/OB for children with disabilities with 2–3 h of screentime (aOR = 1.32, 95% CI: 0.97, 1.80), but these findings were again not significant (*p* ≥ 0.05).

With respect to family resilience, associations with childhood OW/OB varied by resilience level and timepoint (Figure 1; Table 2: Models 1 and 2). At pre-COVID, relative to children with high resiliency scores, those with medium family resilience exhibited lower odds of OW/OB (aOR = 0.90, 95% CI: 0.75, 1.06), after adjustment for relevant covariates and screentime; this association was not statistically significant (*p* ≥ 0.05). During COVID, estimates from Model 2 indicate that the adjusted odds for the medium resiliency group remained largely unchanged (aOR = 0.85, 95% CI: 0.71, 1.01, *p* ≥ 0.05). In contrast, children with low resiliency scores had significantly higher adjusted odds of being OW/OB at pre-COVID compared with their high resilience peers (aOR = 1.31, 95% CI: 1.05, 1.65, *p* < 0.05). As shown in Model 2, the adjusted odds for children with low family resilience decreased during COVID to approximately 1.0 and were no longer statistically significant (aOR = 0.99, 95% CI: 0.77, 1.28, *p* ≥ 0.05).

In the subgroup of children with developmental disabilities, associations between family resilience and OW/OB were minimal, with all adjusted odds ratios close to 1.0 and none statistically significant (Figure 2; Table 2: Models 3 and 4). Pre-COVID results indicate that children with medium resiliency scores had slightly higher odds of OW/OB compared with those with high resilience (aOR = 1.02, 95% CI: 0.79, 1.33), after adjusting for covariates and screentime use; in the during COVID model, these adjusted odds decreased to 0.97 (95% CI: 0.75, 1.25). Neither of these associations were significant (pre-COVID: *p* ≥ 0.05, during COVID: *p* ≥ 0.05). Similarly, relative to children with high family resilience, those with low resiliency demonstrated higher adjusted odds in the pre-COVID model (aOR = 1.05, 95% CI: 0.77, 1.44), which decreased to below 1.0 during COVID (aOR = 0.89, 95% CI: 0.64, 1.24). These associations also did not achieve statistical significance (pre-COVID: *p* ≥ 0.05, during COVID: *p* ≥ 0.05).

Notably, both pre and during the pandemic, age was a significant factor associated with OW/OB among all children and the subgroup with developmental disabilities. For both time periods, children aged 10–12 had significantly higher odds of experiencing OW/OB when compared to their 15–17-year-old peers, after controlling for screentime, family resiliency, and other covariates (pre-COVID: aOR = 1.47, *p* < 0.001; during COVID: aOR = 1.62, *p* < 0.001) (Figure 1; Table 2: Models 1 and 2). Across both periods, adjusted odds of OW/OB trended closer to 1.0 for all children aged 12–15 years relative to those aged 15–17 years. A similar age-related pattern was observed within the subgroup of children with developmental disabilities in both periods, with higher adjusted odds ratios of OW/OB among children aged 10–12 (pre-COVID: aOR = 1.54, *p* < 0.001; during COVID: aOR = 1.25, *p* < 0.05) and attenuated associations for those aged 12–15 (pre-COVID: aOR = 1.22, *p* ≥ 0.05; during COVID: aOR = 0.96, *p* ≥ 0.05) (Figure 2; Table 2: Models 3 and 4).

## 4. Discussion

In this study, we examined screentime and family resiliency as potential risk and protective factors for childhood OW/OB both pre- and during the COVID-19 pandemic. We conducted two parallel analyses, one including all children and one restricted to the subgroup with developmental disabilities, to compare patterns of associations with OW/OB within the two populations. Although multiple studies have examined the rate of childhood OW/OB during COVID-19 [1,2], our focus was on investigating trends in OW/OB specifically for children with developmental disabilities during the pandemic due to a gap in the literature on this topic.

Our findings demonstrated that the prevalence of OW/OB among all children rose during the COVID-19 pandemic. Specifically, we observed an absolute weighted increase of 3.0% (from 32.8% pre-COVID to 35.8% during COVID) in the cohort of all OW/OB children. While this trend aligns with pandemic-related weight gain patterns reported in prior studies, the magnitude of increase appeared less pronounced than the dramatic acceleration in weight gain described in some contemporaneous reports [1,11]. This discrepancy may be due to the NSCH not including BMI prevalence for children age two to under 10 years of age.

The prevalence of OW/OB also increased within the developmental disabilities subgroup during the pandemic, rising by 1.9% (37.4% pre-COVID, 39.3% during COVID). As children in this subgroup already faced elevated baseline risk factors for OW/OB, such as prescription medication side effects and genetic predisposition [8], a more pronounced increase in the proportion of OW/OB individuals was expected due to the compounding effects of pandemic-related conditions like social distancing and stay-at-home orders [14,24]. It is possible that this stark of an effect was not observed because we used cross-sectional pandemic data across two years and the effects of OW/OB had a later onset.

We also observed that the majority of OW/OB children, including all children and those in the developmental disabilities subgroup, reported at least 2 h of daily screentime use pre-pandemic and during the pandemic. Of note, the proportion of children reporting ≥4 h of screentime use increased during COVID for both cohorts, an observation that is unsurprising given the greater social isolation reported during the pandemic [14,24]. Additional analyses that accounted for family resiliency, demographics, socioeconomic characteristics, and environmental variables showed positive associations between screentime and the adjusted odds of OW/OB for all children in both the 2–3 h and ≥4 h groups pre- and during the pandemic—findings that are in line with other study reports of positive associations between screentime and OW/OB status [16,17].

Interestingly, although screentime served as a risk factor for OW/OB in the all children cohort, associations between screentime and the adjusted odds of OW/OB were markedly weaker among children in the developmental disabilities’ subgroup pre- and during-COVID. This deviation from our initial hypothesis may be due to the type of respondents that the NSCH was able to reach. However, OW/OB children with developmental disabilities showed a 9.5% increase (39.9% to 49.4%) in ≥4 h screentime use during the pandemic, indicating that screentime use has markedly increased during the pandemic for this subgroup.

Overall, descriptive statistics indicated that family resiliency was generally prevalent and increased during the pandemic for all children with OW/OB as well as for those with developmental disabilities. Associations between family resiliency and OW/OB, however, were mostly weak, except in the pre-pandemic period among all children, where children with lower family resiliency had significantly higher odds of OW/OB than those with higher resiliency. Therefore, while we anticipated a negative association, and thus protective effect, between family resilience and OW/OB to be observed consistently across cohorts and COVID time periods, our results generally did not support this hypothesis. This is line with results from other research work using 2016 NSCH data that found no significant association between family resiliency and childhood OW/OB [32]. It is thus possible that the non-significant results for family resiliency associations observed in our study stem from the complex nature of this variable and its interactions with other stressors such as adverse childhood experiences [23]. Hence, although some studies have noted the positive influence of family resiliency on children’s overall health and OW/OB status [22,23], other research has suggested that family resiliency is a plausible yet complex factor in childhood OW/OB, warranting further investigation to clarify its role [21,22].

One additional finding of this study was the clear association between age and OW/OB status which aligns with evidence in existing literature. We found that across both pre- and during-COVID periods, in both all children and the subgroup with developmental disabilities, the youngest children (ages 10–12) had consistently higher odds of OW/OB compared with the oldest children (ages 15–17). These results are consistent with findings from Lange et al. [11] but they differ slightly from other studies that point to higher prevalence in older aged children [1,6]. The inconsistency in findings could be due to parent reports of their child’s BMI in the NSCH dataset. However, these findings underscore the importance of focusing obesity prevention and intervention efforts on younger children as key strategies in reducing pediatric OW/OB.

There are several limitations to this work. First, post hoc adjustment for multiple comparisons was not performed; therefore, statistically significant findings should be interpreted with caution. We report odds ratio estimates and 95% confidence intervals to convey the magnitude and precision of associations beyond *p*-values. Secondly, NSCH captures cross-sectional, self-reported data. The survey data is thus likely subject to bias based on the nature and type of respondents that the NSCH was able to reach during COVID, including nonresponse and recall biases. Additionally, as a result of variable measurement constraints, we might be missing information about important components of screentime, family resilience, and BMI. As BMI is not collected for children aged 2 to under 10 years in the NSCH, important insights into weight trends among these children may be overlooked.

Research has also shown that parents have a self-report bias when reporting height and weight of their children [33]. Specifically, parents often underestimate weight and overestimate height measurements for their children, which could influence BMI status. This trend of parental over or underestimation of their child’s height and weight is also most likely reflected in the family resiliency measure due to social desirability biases. Families are more likely to identify as high resiliency as compared to families self-identifying as low resiliency [34].

In light of the age and screen time associations observed in our study, it is important that these anti-obesity public health interventions be adapted to meet the specific needs of children with developmental disabilities particularly with the negative impact of the COVID-19 pandemic using culturally sensitive strategies [35]. Although family resiliency did not emerge as a significant protective factor for OW/OB during COVID for all children and children with developmental disabilities in our analysis, however there is a gap in evidence around applying family resiliency as a preventive mechanism in obesity prevention efforts. Additional research exploring the complex relationship between family resilience and childhood OW/OB is needed at this time to inform future obesity prevention program interventions and policy decisions.

## 5. Conclusions

The prevalence of overweight/obesity among children overall and for those with developmental disabilities continued to increase during the COVID-19 pandemic. Study results point to screentime as a key determinant impacting OW/OB status among children and children with developmental disabilities during the pandemic period. Further research on applying the protective role of family resiliency on screentime use and childhood obesity may provide insights into developing best practices and tailored interventions in supporting children with early OW/OB screening and programs tailored towards the youngest group of children aged 10–12 years or below.

## Figures and Tables

**Table 1:** Weighted Descriptive Statistics for All Children (With or Without Disabilities) and for Children with Developmental Disabilities Identified as Overweight/Obese Pre- and During COVID-19.

Characteristic	All Children		Children with DD	
	Pre-COVID Weighted % (n = 26,205 ^1^; Wt. n = 4,403,706 ^2^) (32.8% ^3^)	During COVID Weighted % (n = 35,368 ^1^; Wt. n = 4,752,235 ^2^) (35.8% ^3^)	Pre-COVID Weighted % (n = 7572 ^1^; Wt. n = 1,282,728 ^2^) (37.4% ^3^)	During COVID Weighted % (n = 11,024 ^1^; Wt. n = 1,482,444 ^2^) (39.3% ^3^)
**Screentime (Hours)**				
<1–1.5	13.9	10.1	13.0	8.6
2–3	52.6	48.3	47.1	41.9
≥4	33.5	41.6	39.9	49.4
**Family Resilience**				
Low	9.7	6.9	12.5	9.5
Medium	12.0	11.1	17.1	15.5
High	78.3	82.0	70.4	74.9
**Health Insurance Type**				
Private Only	51.1	48.4	45.7	43.3
Public Only	37.5	37.5	40.5	44.0
Public and Private	4.8	5.9	8.6	7.1
Uninsured	6.7	8.2	5.3	5.5
**Care Met Medical Home Criteria**				
Yes	43.2	42.5	38.8	40.8
No	56.8	57.5	61.2	59.2
**Household Income Level**				
0–99% FPL	22.4	21.6	25.3	23.0
100–199% FPL	27.0	26.2	26.5	24.2
200–399% FPL	28.2	29.7	26.9	31.5
≥400% FPL	22.4	22.4	21.2	21.4
**Parents’ Education**				
Less Than High School	15.0	13.0	8.8	10.4
High School Degree/GED	24.2	25.5	26.7	24.9
Some College/Technical	25.0	24.1	29.7	25.9
College Degree or Higher	35.8	37.4	34.8	38.8
**Family Structure**				
Two Parents, Currently Married	55.7	57.0	50.9	52.8
Two Parents, Not Currently Married	9.1	7.5	9.4	7.8
Single Parent (Mother/Father)	29.0	29.5	31.7	30.5
Grandparent/Other Family	6.2	6.0	8.1	9.0
**Age (Years)**				
10–12	41.4	42.5	39.8	38.4
12–15	36.1	35.7	37.4	37.2
15–17	22.5	21.8	22.7	24.4
**Race/Ethnicity**				
Non-Hispanic White	44.5	42.2	53.1	49.1
Non-Hispanic Black	17.3	15.6	16.9	13.6
Hispanic	30.5	33.5	23.3	28.9
Asian	2.4	3.1	0.7	0.9
AI/AN, Native Hawaiian, PI	0.7	0.7	0.5	0.6
Multi-Race/Other	4.6	4.9	5.4	6.8
**Sex**				
Male	54.4	56.1	59.5	60.6
Female	45.6	43.9	40.5	39.4
**Neighborhood Amenities**				
No Amenity	12.7	12.2	12.6	12.2
One Amenity	12.5	13.0	12.3	12.8
Two Amenities	19.5	18.5	18.2	20.1
Three Amenities	20.4	22.9	24.6	24.1
Four Amenities	35.0	33.3	32.4	30.8
**Household Food Situation**				
Always Had Nutritious Food	58.1	61.4	49.1	54.0
Afford to Eat, Not Always Nutritious	34.2	32.7	38.9	36.4
Sometimes/Often Could Not Afford	7.7	5.9	11.9	9.6
**Health Status**				
Excellent/Very Good	81.9	79.9	71.0	66.6
Good	14.6	16.8	21.3	26.2
Fair/Poor	3.6	3.3	7.7	7.1

DD = Developmental disabilities. ^1^ Unweighted sample count. ^2^ Weighted sample count. ^3^ Weighted percentage of the total sample classified as OW/OB.

**Table 2:** **Adjusted Weighted Regression Results for Factors Associated with Overweight or Obesity Among All Children and Children with Developmental Disabilities Pre- and During COVID Years.** Summary of the multivariate logistic regression models fit to assess associations between screentime and OW/OB and between family resilience and OW/OB for all children (with or without developmental disabilities and among the subgroup of children with developmental disabilities. Results are reported as adjusted odds ratios (aOR) with 95% confidence intervals (CI). References groups are denoted with a “(ref)” label where appropriate.

Characteristic	All Children		Children with DD	
	Model 1: Pre-COVID aOR (95% CI)	Model 2: During COVID aOR (95% CI)	Model 3: Pre-COVID aOR (95% CI)	Model 4: During COVID aOR (95% CI)
**Screentime (Hours)**				
<1–1.5 (ref)	-	-	-	-
2–3	1.21 * (1.01, 1.45)	1.40 ^†^ (1.18, 1.66)	0.90 (0.66, 1.23)	1.32 (0.97, 1.80)
≥4	1.32 ^§^ (1.09, 1.61)	1.49 ^†^ (1.24, 1.78)	1.15 (0.83, 1.59)	1.39 * (1.02, 1.89)
**Family Resilience**				
High (ref)	-	-	-	-
Low	1.31 * (1.05, 1.65)	0.99 (0.77, 1.28)	1.05 (0.77, 1.44)	0.89 (0.64, 1.24)
Medium	0.90 (0.75, 1.06)	0.85 (0.71, 1.01)	1.02 (0.79, 1.33)	0.97 (0.75, 1.25)
**Health Insurance Type**				
Private (ref)	-	-	-	-
Public Only	1.16 (0.95, 1.41)	1.21 * (1.04, 1.41)	0.97 (0.72, 1.32)	1.30 * (1.02, 1.64)
Public and Private	1.12 (0.85, 1.47)	1.34 * (1.04, 1.74)	1.18 (0.81, 1.71)	1.19 (0.86, 1.64)
Uninsured	0.80 (0.60, 1.07)	1.09 (0.85, 1.40)	1.11 (0.70, 1.78)	0.97 (0.61, 1.55)
**Care Met Medical Home Criteria**				
Yes (ref)	-	-	-	-
No	1.00 (0.88, 1.13)	1.01 (0.91, 1.13)	1.07 (0.87, 1.30)	0.96 (0.81, 1.13)
**Household Income Level**				
≥400% FPL (ref)	-	-	-	-
0–99% FPL	1.07 (0.85, 1.36)	1.13 (0.93, 1.39)	1.30 (0.91, 1.87)	1.21 (0.88, 1.66)
100–199% FPL	1.20 (0.98, 1.47)	1.20 * (1.01, 1.42)	1.23 (0.88, 1.70)	1.00 (0.75, 1.32)
200–399% FPL	1.14 (1.00, 1.31)	1.18 * (1.04, 1.34)	1.25 (0.99, 1.59)	1.17 (0.94, 1.46)
**Parents’ Education**				
College Degree or Higher (ref)	-	-	-	-
Less Than High School	1.65 ^§^ (1.18, 2.30)	1.69 ^†^ (1.27, 2.26)	1.35 (0.79, 2.32)	2.21 ^§^ (1.26, 3.89)
High School Degree/GED	1.61 ^†^ (1.34, 1.93)	1.61 ^†^ (1.36, 1.89)	1.66 ^§^ (1.19, 2.32)	1.49 ^§^ (1.16, 1.92)
Some College/Technical	1.32 ^†^ (1.15, 1.53)	1.40 ^†^ (1.22, 1.60)	1.36 * (1.07, 1.72)	1.38 ^§^ (1.12, 1.70)
**Family Structure**				
Two Parents, Currently Married (ref)	-	-	-	-
Two Parents, Not Currently Married	1.11 (0.85, 1.45)	1.06 (0.82, 1.38)	1.20 (0.78, 1.83)	0.98 (0.67, 1.45)
Single Parent (Mother/Father)	1.07 (0.92, 1.26)	1.11 (0.97, 1.27)	1.08 (0.85, 1.38)	1.02 (0.82, 1.26)
Grandparent/Other Family	0.91 (0.65, 1.28)	1.14 (0.88, 1.47)	1.07 (0.72, 1.61)	1.23 (0.83, 1.80)
**Age (Years)**				
15–17 (ref)	-	-	-	-
10–12	1.47 ^†^ (1.25, 1.73)	1.62 ^†^ (1.42, 1.85)	1.54 ^†^ (1.21, 1.97)	1.25 * (1.01, 1.57)
12–15	1.14 (0.97, 1.33)	1.07 (0.93, 1.22)	1.22 (0.95, 1.57)	0.96 (0.77, 1.20)
**Race/Ethnicity**				
Non-Hispanic White (ref)	-	-	-	-
Non-Hispanic Black	1.59 ^†^ (1.31, 1.93)	1.41 ^†^ (1.19, 1.66)	1.47 * (1.06, 2.03)	1.13 (0.86, 1.49)
Hispanic	1.30 ^§^ (1.09, 1.55)	1.48 ^†^ (1.26, 1.72)	1.25 (0.92, 1.71)	1.38 * (1.05, 1.80)
Asian	0.54 ^†^ (0.39, 0.76)	0.77 * (0.61, 0.97)	0.40 * (0.19, 0.87)	0.71 (0.38, 1.34)
AI/AN, Native Hawaiian, PI	1.60 * (1.06, 2.40)	1.06 (0.68, 1.67)	0.85 (0.41, 1.73)	0.76 (0.39, 1.47)
Multi-Race/Other	1.06 (0.85, 1.32)	1.16 (0.95, 1.41)	1.07 (0.76, 1.51)	1.32 (0.95, 1.82)
**Sex**				
Male (ref)	-	-	-	-
Female	0.80 ^†^ (0.70, 0.90)	0.66 ^†^ (0.60, 0.74)	1.00 (0.82, 1.21)	0.76 ^§^ (0.64, 0.91)
**Neighborhood Amenities**				
Four Amenities (ref)	-	-	-	-
No Amenity	1.30 ^§^ (1.08, 1.56)	1.40 ^†^ (1.19, 1.64)	1.26 (0.92, 1.73)	1.27 (0.97, 1.68)
One Amenity	1.25 * (1.02, 1.55)	1.15 (0.95, 1.41)	0.98 (0.71, 1.35)	1.31 (0.96, 1.79)
Two Amenities	1.27 * (1.05, 1.52)	1.07 (0.92, 1.24)	1.06 (0.78, 1.44)	1.06 (0.83, 1.35)
Three Amenities	0.98 (0.83, 1.16)	1.06 (0.91, 1.22)	1.16 (0.90, 1.51)	1.10 (0.87, 1.39)
**Household Food Situation**				
Always Had Nutritious Food (ref)	-	-	-	-
Afford to Eat, Not Always Nutritious	1.34 ^†^ (1.16, 1.56)	1.21 ^§^ (1.06, 1.38)	1.53 ^†^ (1.20, 1.95)	1.23 * (1.00, 1.51)
Sometimes/Often Could Not Afford	1.35 * (1.01, 1.80)	1.28 (0.95, 1.73)	1.48 (0.98, 2.23)	1.53 * (1.04, 2.25)
**Health Status**				
Excellent/Very Good (ref)	-	-	-	-
Good	1.96 ^†^ (1.58, 2.44)	2.21 ^†^ (1.84, 2.65)	1.67 ^†^ (1.30, 2.16)	2.05 ^†^ (1.62, 2.60)
Fair/Poor	2.51 ^†^ (1.47, 4.27)	2.47 ^†^ (1.48, 4.13)	2.39 ^†^ (1.49, 3.83)	2.76 ^†^ (1.76, 4.34)

DD = Developmental disabilities. ^†^ *p* < 0.001, ^§^ *p* < 0.01, * *p* < 0.05.

## Data Availability

The data presented in this study are available on request from the corresponding author.
